# Supplementary material for: O Desafio da Profilaxia Eficaz do Acidente Vascular Cerebral Secundário em Pacientes Chagásicos
Source: Arq Bras Cardiol. 2024 Jun 14;121(5):e20240008. [Article in Portuguese] doi: 10.36660/abc.20240008 (PMC11216335; doi:10.36660/abc.20240008)
Supplement: Translation

# Translation: The Challenge of Effective Secondary Stroke Prophylaxis in Chagas Patients

**DOI:** 10.36660/abc.20240008i

**Authors:** Vinícius Viana Abreu Montanaro

**Affiliations:** 1 Brasília Brazil Rede SARAH de Hospitais de Reabilitação, Brasília – Brazil

**Keywords:** Secondary, Stroke, Prophylaxis, Chagas

The recent update on the guideline for the management of cardiomyopathy in Chagas disease^1^ brings several significant improvements. The evidence on etiological stroke investigation, management of secondary prophylaxis, and overall epidemiology are all of vital importance for clinicians to improve treatment for these patients.

One concern that arises from the guideline pertains to the indication of oral anticoagulation, particularly the suggestion that any previous ischemic stroke would warrant such intervention.^1^

Through epidemiological studies, we now understand that the prevalence of non-cardioembolic strokes, such as large vessel or small vessel diseases, is significant.^2,3^ There is also evidence that atherosclerotic causes of stroke have the choice of antiplatelets as secondary prophylaxis.^4^ In several cohorts, the presence of cardiac alterations in patients with Chagas disease and stroke may not be overt, casting doubt on the benefit of oral anticoagulation in all patients with a previous stroke.^2,3,5^

A more appropriate and evidence-based recommendation would be the use of oral anticoagulants in patients with a previous stroke deemed cardioembolic via the etiological classification systems currently available.^2,5^ This approach would ensure that the treatment is tailored to the specific needs of the patient, thereby maximizing the potential benefits and minimizing unnecessary risks.

Moreover, it is crucial to consider the individual patient's risk factors and comorbidities when deciding on the best course of treatment. For instance, patients with a high risk of bleeding may not be suitable candidates for anticoagulation therapy. Therefore, a comprehensive risk-benefit analysis should be conducted for each patient before initiating secondary stroke prophylaxis.

In conclusion, while the updated guideline provides valuable insights, it is essential to apply these recommendations judiciously, considering the unique circumstances of each patient. Further research is needed to refine these guidelines and ensure optimal patient outcomes.
